# The Practice of Physical Activity in the Setting of Lower-Extremities Sarcomas: A First Step toward Clinical Optimization

**DOI:** 10.3389/fphys.2017.00833

**Published:** 2017-10-25

**Authors:** Mohamad Assi, Mickael Ropars, Amélie Rébillard

**Affiliations:** ^1^EA1274 Laboratory “Movement, Sport and Health Sciences” M2S, University of Rennes 2-ENS Rennes, Bruz, France; ^2^Orthopedic and Trauma Surgery Unit-Hugortho Pontchaillou University Hospital, Rennes, France

**Keywords:** cancer, lower-extremities sarcomas, quality of life, rehabilitation, physical activity, exercise

## Abstract

Lower-extremities sarcoma patients, with bone tumor and soft-tissue sarcoma, are a unique population at high risk of physical dysfunction and chronic heart diseases. Thus, providing an adequate physical activity (PA) program constitutes a primary part of the adjuvant treatment, aiming to improve patients' quality of life. The main goal of this paper is to offer clear suggestions for clinicians regarding PA around the time between diagnosis and offered treatments. These preliminary recommendations reflect our interpretation of the clinical and preclinical data published on this topic, after a systematic search on the PubMed database. Accordingly, patients could be advised to (1) start sessions of supportive rehabilitation and low-intensity PA after surgery and (2) increase PA intensities progressively during home stay. The usefulness of PA during the preoperative period remains largely unknown but emerging preclinical data on mice bearing intramuscular sarcoma are most likely discouraging. However, efforts are still needed to in-depth elucidate the impact of PA before surgery completion. PA should be age-, sex-, and treatment-adapted, as young/adolescent, women and patients receiving platinum-based chemotherapy are more susceptible to physical quality deterioration. Concerning PA intensity, the practice of moderate-intensity resistance and endurance exercises (30–60 min/day) are safe after surgery, even when receiving adjuvant chemo/radiotherapy. The general PA recommendations for cancer patients, 150 min/week of combined moderate-intensity endurance/resistance exercises, could be feasible after 18–24 months of rehabilitation. We believe that these suggestions will help clinicians to design a low-risk and useful PA program.

## Introduction

Main concerns regarding sarcoma essentially consist in optimizing treatments, general outcomes and survival rates. Today, PA constitutes an essential part of the primary anticancer therapy, due to its ability to reduce treatment-related adverse side-effects, maintain muscle performance, and enhance recovery (Courneya et al., 2011; Friedenreich et al., 2016a). Regarding PA in lower-extremities sarcoma patients (LESP), the few existing studies focused on clinical measurement of physical functioning after LESP treatments and/or limb salvage (Gundle et al., 2014, 2015). There is certainly an insufficient interest in PA application throughout sarcoma treatment and a lack of information regarding pre, per, and postoperative optimal level of PA for sarcoma patients. This is particularly the case in LESP, where general mechanical alteration occurs due to tumor growth, surgical treatment and its inherent muscular sacrifice, which impairs patients' physical functioning (Morrison, 2003; Tang et al., 2012; Mason et al., 2013). Additionally, lower-extremities sarcomas are highly frequent with possible natural vascular pathogenesis linked to this particular localization (Nishinari et al., 2015; Poultsides et al., 2015); thus, requiring heavy interventions and increasing the risk of physical quality deterioration. After the announcement of such diagnosis and its potential treatments, most of patients, particularly young and active ones ask for the level of PA to preserve before surgery or adjuvant chemotherapy. Such information is crucial in order to maintain overall PA, without impairing general outcomes and survival. This paper is a first attempt to provide general PA recommendations for LESP. These suggestions reflect the current progress regarding the practice of PA in sarcoma at both clinical and preclinical levels.

## Methodology

We performed a systematic search on the PubMed database, until August 2017, in the aim to identify clinical and preclinical studies that addressed the impact of PA in sarcoma. The search was performed with a combination of the following keywords: (1) “Sarcoma” and “Exercise,” (2) “Sarcoma” and “Physical Activity” and (3) “Sarcoma,” “Exercise,” and “Quality of Life.” The initial search identified 124 clinical study and five preclinical studies. Concerning clinical papers, after examination of the abstract and removal of duplicates, we obtained 21 clinical papers addressing the usefulness of PA in sarcoma. After reviewing of full-text, we excluded nine papers, such as, psychological reports and studies describing preliminary data. There was no restriction concerning the number of patients or the date at which the study was performed. Finally, we obtained 12 study: seven studies performed on LESP and five studies performed on sarcoma in general without providing separated data for LESP. Among these, nine studies have assessed the impact of sarcomas and/or their treatments on physical function and quality of life (QoL), and only three studies have determined the effect of direct PA intervention and rehabilitation in sarcoma patients (Supplementary Table 1). The molecular findings of preclinical studies that tested different modalities of PA in animals bearing intramuscular, subcutaneous or intra-peritoneal sarcoma tumors are listed in Supplementary Table 2.

## Alteration of physical performance in cancer patients: a focus on lower-extremities sarcomas

Despite the reduced physical ability observed in many cancers, patients with primary malignant bone tumors (e.g., osteosarcoma, chondrosarcoma, Ewing sarcoma, and osteoblastoma) and soft-tissue sarcomas (e.g., liposarcoma) in the lower-extremities, constitute a unique population that manifests a severe deterioration of physical quality following limb-spared surgery or amputation (Ness et al., 2005). Affected gait and postural automatism, reduced walking velocity and cadence, weak lower-extremities strength, limited performance, and restricted ability to attend school or work, are the most reported consequences of simple and complex surgery in childhood and adult survivors of lower-extremities sarcomas (Ness et al., 2005; Gerber et al., 2006; Hoffman et al., 2013). Other studies have shown that patients treated for lower-extremities sarcomas spend 54% of their time sitting and present severe symptoms of fatigue and anxiety (Rosenbaum et al., 2008). Those patients may exhibit poor ambulatory PA, since the daily count of walking steps and time spent in activity do not meet PA guidelines (Wampler et al., 2012). Some data indicate that patients perform a median number of ~4,500 steps per day, which is much lesser than the recommendations of the world health organization (WHO) (10,000 steps/day) (Rosenbaum et al., 2008; WHO, 2010; Sheiko et al., 2012).

**Figure 1:**
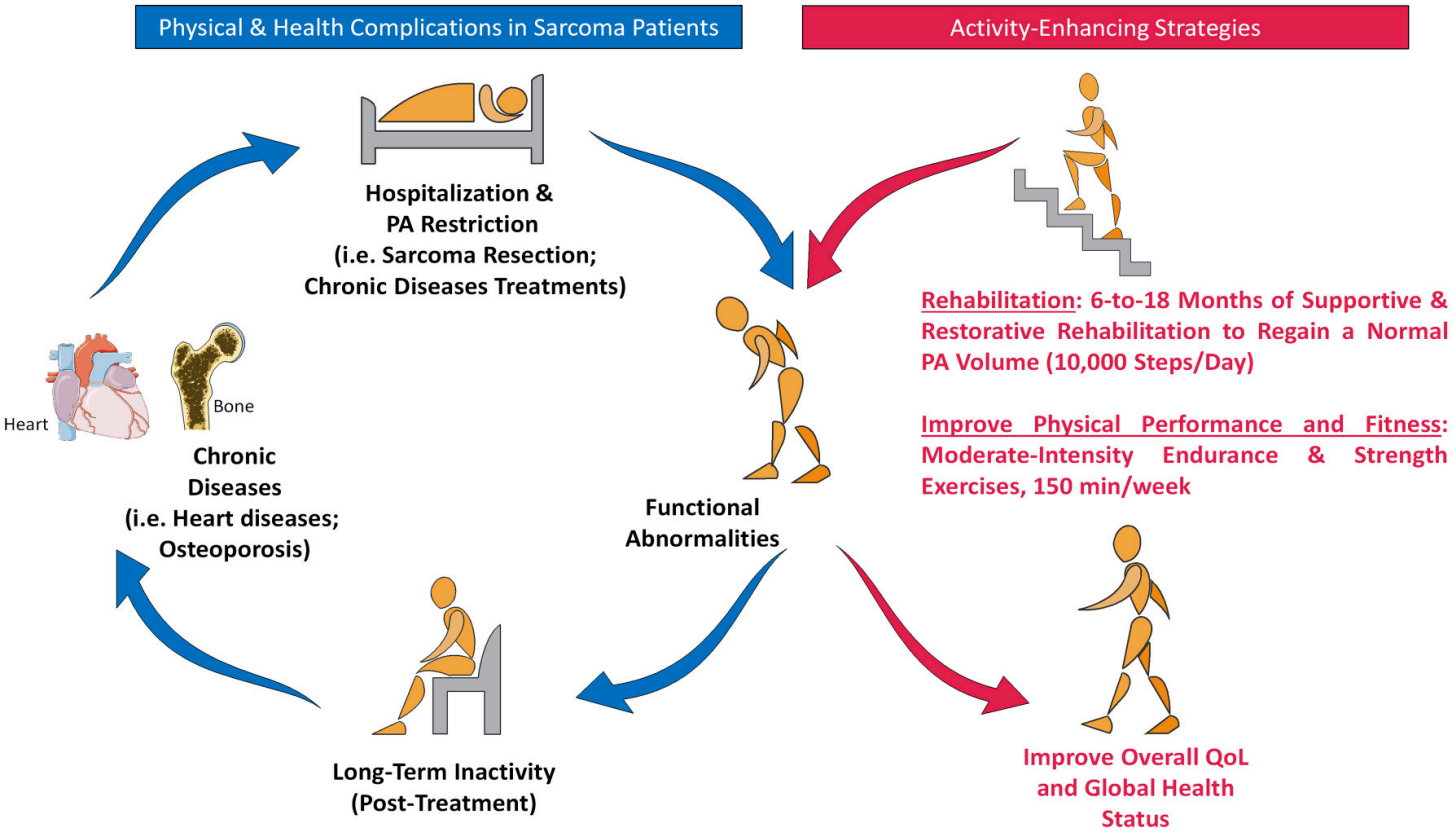
Physical dysfunction in lower-extremities sarcoma patients and the potential role of physical exercise. The heavy surgical intervention that undergo lower-extremities sarcoma patients leads to functional abnormalities and reduces autonomy. The operated patient suffers from severe fatigue, which could lead to long-term physical inactivity and overweight. Subsequently, this unique population is highly susceptible to the development of chronic illnesses, which extends the hospitalization period and restricts PA practice. Intervention with moderate-intensity PA could break this vicious circle and improve patient's QoL. PA, Physical Activity; QoL, Quality of Life.

Diagnosed as child, adolescent, or adult, the sedentary behavior after sarcoma treatment may place patients at high risk of other chronic illnesses like, cardiovascular dysfunction, diabetes, and kidney failure (Bobowski and Baker, 2016). Experienced clinicians note that the presence of heart diseases signs at 30-year-old is usually rare, while it is commonly found in treated sarcoma patients (Bobowski and Baker, 2016). Fortunately, these conditions could be modifiable by lifestyle changes, including diet and exercise. Indeed, exercise-oncology studies performed mainly on breast and prostate cancer during/after treatment have clearly demonstrated that moderate-intensity PA reduces fatigue and social physical anxiety and improves cardiorespiratory functions (Milne et al., 2008; Galvao et al., 2010). Importantly, the finding that sarcoma patients meeting PA guidelines exhibit better QoL, has stressed the importance of physical exercise in this population (Murnane et al., 2015). From this perspective, PA could be proposed for LESP to reduce physical disability and aid them to regain a healthy lifestyle (Figure 1).

## Physical activity as a strategy to improve the QoL of patients treated for lower-extremities sarcomas

### Frequency, intensity, type, and time (FITT) of exercise

Endurance and resistance are the two types of exercise that could be prescribed for cancer patients. Cancer patients may at least practice 150 min per week of moderate-intensity endurance exercise or endurance combined to resistance exercise (Schmitz et al., 2010; INC, France, 2017). Combination of endurance and resistance exercises, mainly tested in breast and prostate cancer patients, seem to improve QoL, physical fitness, muscle strength, and lean body mass and decrease systemic inflammation (Milne et al., 2008; Galvao et al., 2010). However, endurance exercise could have a wider applicability in cancer, as patients show a trend to engage in significantly more PA in endurance training comparing to resistance exercise (Santa Mina et al., 2013). This could be due to cancer-related fatigue and muscle weakness, making endurance exercise easier to practice and suitable to patients' situation. Therefore, convincing evidences exist about the beneficial effects of endurance exercise alone or in combination with resistance exercise in cancer survivors.

Given the heavy surgical procedure that directly affects the motility of sarcoma patients, a program of supportive rehabilitation would be necessary during the first weeks post-treatment. Indeed, positioning treatment, relaxation therapy, breathing training, walking, and flexibility exercises are safe and feasible in 93% of patients with advanced stages of cancer, including sarcomas (Jensen et al., 2014). Gentle massage for lymphedema treatment are among the most practiced exercises by sarcoma patients (Jensen et al., 2014). After palliative and supportive rehabilitation, where patients' independency starts to increase as well as their QoL, a combination of moderate endurance and resistance exercises are needed to restore physical function. These types of exercise have not been widely tested in sarcoma, only one study have shown that endurance and resistance exercises are safe in LESP after surgery (Winter et al., 2013). Sarcoma patients prefer to be in direct contact with an exercise physiologist with experience in oncology or a fitness expert (Zebrack, 2008; Zebrack et al., 2008; Belanger et al., 2012). This indicates that patients are aware of their unique case that requires a particular follow-up and special recommendations; thus, every effort should be made to provide those patients with the appropriate team care. Exercise professionals are the best qualified for this mission, discussing with patients to set a short- and long-term goals prior to starting exercise, as this could reduce patients' fear from exercise and increase long-time adherence during home stay (Murnane et al., 2015). Exercise program should be designed on a single-patient basis (Sasso et al., 2015). A moderately intense endurance exercise could be prescribed to improve cardiorespiratory fitness (Convertino, 2007) and a moderate-to-high intensity endurance exercise in combination with resistance exercise sessions could be given to strengthen skeletal muscles and reduce fatigue (MacDougall et al., 1998). The prescription of exercise must be also adapted to the age of the patient. For adults, 30 min of moderate-to-high intensity endurance exercise could be performed 5 days weekly in association with 2 days of strengthening exercises. While, for child, 60 min of moderate-to-high intensity exercise should be practiced 7 days per week (Centers for Disease Control and Prevention, 2008).

### Starting point of intervention: during sarcoma treatment and/or post-treatment

Clinical studies addressing the impact of PA in LESP are performed during and/or post-treatment. Recently, Müller and coworkers addressed the impact of a 4-week rehabilitation program consisting of land-based and aquatic exercises in 26 sarcoma patients without a control group. After 6-month post-rehabilitation, patients exhibited a significant increase in PA volume and cadence, concomitantly with an important improvement in endurance and PA intensity (Muller et al., 2016). In an open non-randomized clinical trial, Winter and colleagues have studied a population of 31 young patients diagnosed with osteosarcoma in the distal/proximal femur and proximal tibia, which have received an endoprosthetic replacement and then treated with adjuvant chemotherapy and radiotherapy (Winter et al., 2013). Sixteen patients have performed 30–60 min of endurance/resistance exercise sessions in combination with adjuvant therapies during inpatient stay (5 days/stay) and another 15 non-exercising patients that have received a similar treatment were used as control. After an 18-month follow-up, authors found that the volume and time spent in moderately intense activity increased in both control and exercise groups due to general recovery, nevertheless, these parameters were improved in the exercised group (Winter et al., 2013). Despite the small number of studies and patients, these preliminary findings support a positive effect for PA interventions after surgery, in combination or not with adjuvant chemo/radiotherapy.

These activity-enhancing interventions during inpatient stay seem to increase the ability of patients to practice house-based exercise (Winter et al., 2013), which is extremely important to maintain a good body functioning. LESP need to follow a program of supportive rehabilitation and low-intensity exercise during their hospital stay and/or their first year post-treatment. After 6–18 months of rehabilitation, LESP are supposed to reach a normal volume of PA (10,000 step/day) and, therefore, can start a program of moderate-intensity endurance and resistance exercises to further improve their physical performance. At transitional post-treatment phases (e.g., after surgery before starting rehabilitation and after rehabilitation before starting higher intensities of exercises), LESP can pursue a short period of specialized exercise sessions under the supervision of exercise physiologists to provide advice, guidance, and ensure that LESP can autonomously practice the correct techniques of exercise during home stay. Such support is also essential to help young people to navigate through issues like pain, fatigue and overweight that usually face them in the post-operative period (Jones et al., 2009; Phillips-Salimi and Andrykowski, 2013). For organizational, time, and costs issues, treating physician could review patient's physical improvements, during regular consultations. Additionally, a booklet containing motivational cues, clear exercise recommendations, and simple technical tips could be given for LESP to assist them in their daily PA practice during home stay.

## The impact of physical exercise on cancer progression and survival: clinical evidence

In addition to its role in improving patients' QoL, PA may also delay disease recurrence, which highlights a possible role in the suppression of tumor growth (Meyerhardt et al., 2006b; Ballard-Barbash et al., 2012; Friedenreich et al., 2016a,b). Lahart and colleagues performed a systematic review and meta-analysis in which they included 22 prospective cohort study addressing the effects of PA on breast cancer (Lahart et al., 2015). They found that PA reduce the risk of breast cancer progression in both recurrence and new primaries (Lahart et al., 2015). The findings obtained from Richman et al. in patients with diagnosed prostate cancer, demonstrate the existence of a dose-response relationship between the intensity of practiced PA and the reduction of cancer progression (Richman et al., 2011). Indeed, the delay in prostatic cancer progression is seen in patients who practiced brisk walking for 3 h/week or more, comparing to those engaged in <3 h/week with an easy pace (Richman et al., 2011). Similarly, patients with colon cancer practicing moderate-to-intense activity of 18 metabolic equivalent task (MET)-h/week, exhibit an increase in survival-free recurrence comparing to those practicing 3 MET-h/week (1 MET is the energy that a person expends at rest) (Meyerhardt et al., 2006a; Jeon et al., 2013). Today, breast and prostate cancer patients are advised to increase their levels of PA after diagnosis, as it may have important positive repercussions on tumor growth/evolution and could delay the intervention with conventional anticancer therapies (Friedenreich, 2011).

Unfortunately, PA has not been tested in LESP especially during the preoperative period, between diagnosis and surgery; thus, the effect of increasing activity levels on sarcoma growth/progression remains unknown. There is also a lack of information concerning the adoption of an active lifestyle on overall sarcoma patients' survival and tumor recurrence. However, few preclinical studies have been performed to further understand the impact of an active behavior on tumor evolution in rodents.

## The impact of physical activity on sarcoma growth: preclinical models and molecular mechanisms

In the sarcoma setting, two descriptive studies have tested the impact of forced swimming in mice bearing subcutaneous murine sarcoma-180 tumor (Radak et al., 2001; Sasvari et al., 2011). Authors demonstrated that an 8–10 weeks continuous swimming (1 h/day; 5 days/week; before and after tumor implantation), reduces the size of sarcoma tumors compared to untrained animals, but the molecular mechanisms have not been clearly elucidated (Radak et al., 2001; Sasvari et al., 2011). However, the location of tumors and the chosen modality of exercise are not adapted to draw a relevant conclusion about sarcomas in the lower-extremities. Given the frequent presence of sarcoma within lower-extremities (Peterson et al., 2003) and the absence of information about the usefulness of PA before surgery, it was necessary to start by testing the safety of PA in a preclinical study designed to mimic this clinical context.

Our laboratory has launched a preclinical study aiming to determine the impact of voluntary PA on the growth of liposarcoma (LS) tumor implanted orthotopically in lower-limb skeletal muscles of male nude mice (Assi et al., 2017). Counterintuitively, we observed that active mice exhibited ~1.5 fold increase in tumor mass after 13 weeks (6 weeks before tumor and 7 weeks after tumor) of continuous voluntary running on wheels, compared to mice who ran the half of this duration (6 weeks before tumor; Assi et al., 2017). This is due to higher rates of proliferation and mitosis in tumors of active mice. Based on our *in vivo* and *in vitro* data, we have demonstrated that p38-MAPK and p21 are activated in the tumors of less-active mice and that p38-MAPK is able to up-regulate p21 expression (Assi et al., 2017). We also established a link between the moderate increase in circulating insulin and p38-MAPK/p21 activation.

## Is the practice of exercise disadvantageous during the preoperative period?

As the LS cell line that we injected in mice is derived from a 36-year-old patient, we can suppose that lower-extremities LS tumor will behave similarly in patients in response to PA. This may suggest that increasing PA levels may enhance the growth of tumor in the lower limbs, which complicates the surgical procedure to remove tumor away, increases the risk of amputation/prosthetic replacement and leads to a poorer performance and QoL. However, our preclinical data could be tumor- and context-specific, and future preclinical and clinical studies are still needed to confirm the generality of our conclusion in other types of sarcoma. At the moment, given the incertitude about the role of PA preoperatively, patients could be advised to maintain a normal volume of PA to avoid deconditioning or immobilization, without practicing a supplementary level of PA, like brisk walking. In such case, the patient is not exposed to over-activity and can be autonomous in performing his/her daily tasks. Efforts are still needed to in-depth elucidate the impact of PA before surgery completion, meanwhile, the actual clinical recommendations should be considered as a preventive step against an unproven strategy that could be deleterious for patients' health.

## Conclusion

**Figure 2:**
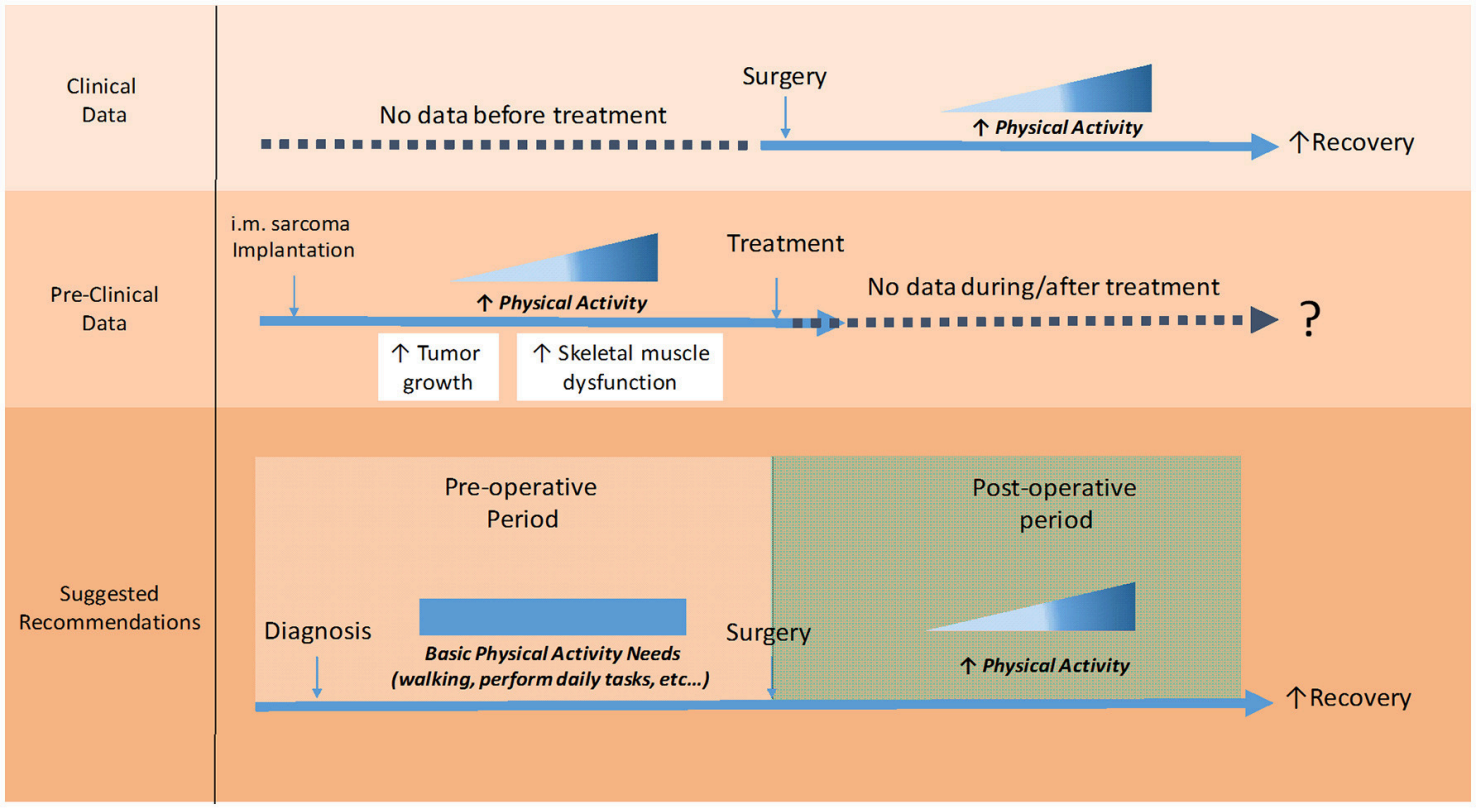
Preliminary recommendations about the time of intervention with physical activity from sarcoma diagnosis until getting home. Existing clinical data indicate that endurance, strength and flexibility exercises are safe and feasible in the post-operative period, as it may aid to improve physical function and recovery. Contrariwise, there is a complete absence of clinical data about the utility of PA in the preoperative period, before tumor excision. Even worse, emerging preclinical data indicate that active mice bearing intramuscular sarcoma, in lower-limbs, exhibited an increase in tumor growth and skeletal muscle dysfunction. Therefore, the combination of available clinical and preclinical data let us suppose that patients could be advised to not increase their levels of PA before surgery, while the volume of PA can rise-up progressively after surgery in combination with a number of rehabilitation sessions. PA, Physical Activity.

The deterioration of QoL in LESP is a fact and the best way to reverse or slow this condition is partly dependent on the prescription of adequate PA interventions. In this paper, we distinguish between two important phases during sarcoma patients' hospitalization, in which PA recommendations could be quite different: (1) the preoperative and (2) post-operative period. Existing clinical data indicate that endurance and strength exercises are safe and feasible during the post-operative period. Contrariwise, there is a complete absence of clinical data about the utility of PA in the preoperative period. Available preclinical data may support a role for PA in promoting intramuscular LS growth and altering skeletal muscle function. As depicted in Figure 2, the combination of the available clinical findings post-operation and preclinical data pre-operation, may give rise to general recommendations. Accordingly, patients could be advised to (1) maintain a normal level of activity without practicing regular exercise, (2) restart sessions of rehabilitation and low-to-moderate intensity PA after surgery and (3) increment PA intensities progressively during home stay. These preliminary recommendations are slightly different from PA guidelines in breast, prostate and colon cancer who are advised to start exercises programs directly after diagnosis. Future studies are still needed to answer an important question: what is the impact of regular or incrementing exercise levels on LESP before undergoing surgery?

## Author contributions

MA and AR designed and planned the work. MA performed systematic search and drafted the original form of the manuscript. AR and MR performed critical revision of manuscript drafts. All authors have read and approved the final manuscript.

### Conflict of interest statement

The authors declare that the research was conducted in the absence of any commercial or financial relationships that could be construed as a potential conflict of interest.

## Supplementary material

Supplementary Table 1 After a systematic search on the PubMed database, this table lists clinical studies assessing the impact of lower-extremities sarcomas and/or their treatments on physical function. Among these, three studies have tested the effect of rehabilitation and physical activity intervention.

Supplementary Table 2 After a systematic search on the PubMed database, this table lists preclinical studies that tested different modalities of PA in animals bearing intramuscular, subcutaneous or intra-peritoneal sarcoma tumors.
