# Supplementary material for: The Practice of Physical Activity in the Setting of Lower-Extremities Sarcomas: A First Step toward Clinical Optimization
Source: Front Physiol. 2017 Oct 25;8:833. doi: 10.3389/fphys.2017.00833 (PMC5660974; doi:10.3389/fphys.2017.00833)
Supplement: Supplementary Table 1 — After a systematic search on the PubMed database, this table lists clinical studies assessing the impact of lower-extremities sarcomas and/or their treatments on physical function. Among these, three studies have tested the effect of rehabilitation and physical activity intervention. [file Table1.pdf]

Reference	Numb. patients	Mean age (years)	Physical activity intervention	Findings
Winter et al. 2013	16 LESP	13.5	30-60 min strength/endurance exercise during inpatient stay	Improve physical capacity during home stays
Hoffman et al. 2013	13 LESP	15.9	-	Weak lower-extremities strength
Wampler et al. 2012	145 LESP	18	-	59% did not meet PA guidelines
Velensek et al. 2008	15 LESP	18		87% risk of cardiac damage
Gerber et al. 2006	13 LESP	16	-	Slow walk velocity and functioncal loss
Azcona et al. 2003	36 LESP	19	-	Low bone mass and osteoporosis
Ness et al. 2005	931 LESP	21		Performance limitation and restricted ability to attend work/school
Ranft et al. 2017	618 Ewing sarcoma	4-to-31	-	Reduced total steps count
Muller et al. 2016	26 sarcoma	11	4-week rehabilitation land and aquatic training	Improve physical activity levels after 6 months follow-up
Murnane et al. 2015	18 sarcoma	23	-	Reduced physical activity and quality of life
Jensen et al. 2014	28 sarcoma	62	Walking, relaxation therapy and positioning treatment	PA was feasible and safe; light exercises for lymph edema were mostly used
Badr et al. 2013	32 sarcoma	1-to-29	-	Increase general and cognitive fatigue; Poor physical appearance
