# Supplementary material for: The Practice of Physical Activity in the Setting of Lower-Extremities Sarcomas: A First Step toward Clinical Optimization
Source: Front Physiol. 2017 Oct 25;8:833. doi: 10.3389/fphys.2017.00833 (PMC5660974; doi:10.3389/fphys.2017.00833)
Supplement: Supplementary Table 2 — After a systematic search on the PubMed database, this table lists preclinical studies that tested different modalities of PA in animals bearing intramuscular, subcutaneous or intra-peritoneal sarcoma tumors. [file Table2.pdf]

Reference	*In vivo* and *in vitro* models	Physical activity intervention	Physical activity impact on sarcoma growth	Physical activity-related molecular changes
Assi et al. 2017	Mice bearing human SW872 sarcoma cells (intramuscular)	Continuous voluntary running on wheels before and after injection (13 weeks)	Increase by 1.5 fold tumor mass; Activity levels correlate with tumor mass	Inhibition of p38 MAPK-p21 pathway within tumor; Decrease circulating insulin levels
Sasvari et al. 2011	Mice bearing murine sarcoma-180 cells (Subcutaneous)	Continuous swimming exercise before and after injection (88 days)	Decrease tumor size by about 50%	Enhance phagocytic activity of peritoneal and spleen macrophages
Radak et al. 2001	Mice bearing murine sarcoma-180 cells (Subcutaneous)	Continuous swimming exercise before and after injection (86 days)	Decrease tumor size by about 43%	-
Japel et al. 1992	Mice bearing murine sarcoma-180 cells (intra-peritoneal)	Continuous treadmill endurance exercise before and after injection (3-to-6 weeks)	No data about tumor growth	Enhance phagocytic activity of peritoneal and spleen macrophages
Lotzerich et al. 1990	Co-incubation of peritoneal macrophages with sarcoma-180 cells	Single exhaustive running session	No data about tumor growth	Increase the cytostatic activity of macrophages
